# Effects of Cold Post-Fermentation Process on Microbial Diversity and Biogenic Amines in Protease-Assisted Fermented *sufu*

**DOI:** 10.3390/foods14050735

**Published:** 2025-02-21

**Authors:** Xiaogang Guo, Kaiyi Chen, Li Chen, Thanh Ninh Le, Minjie Zhao, Haiying Cai

**Affiliations:** 1 School of Biological and Chemical Engineering, Zhejiang University of Science & Technology, Hangzhou 310023, China; guoxgang@outlook.com (X.G.); c18857121265@outlook.com (K.C.); c13096717568@outlook.com (L.C.); 2 Department of Food Science and Engineering, National University of Singapore, Singapore 117542, Singapore; itninh90@gmail.com; 3 College of Biosystems Engineering and Food Science, Zhejiang University, Hangzhou 310058, China; zhaominjie@zju.edu.cn

**Keywords:** *sufu*, low-temperature post-fermentation, biogenic amines, microbial diversity, enzyme-assisted fermentation

## Abstract

This study investigated the effects of enzyme-assisted low-temperature cold fermentation on *sufu*’s microbial diversity, biogenic amine (BA) formation, and physicochemical properties. The results showed that the enzyme-assisted fermentations for both room- and low-temperature groups (RTEF30 and LTEF20, respectively) significantly increased total acid (TA), amino nitrogen (NH3-N), and enzyme activity compared to the non-enzyme fermentation at room-temperature post-fermentation (RTNF30). This indicated that enzyme-assisted fermentation effectively overcame challenges associated with low-temperature fermentation of *sufu*. BA analysis revealed that the LTEF20 group had the highest total BA (3.7 mg/g) and putrescine (1.8 mg/g) levels compared to other groups. Microbial analysis showed that the LTEF20 group exhibited higher microbial diversity compared to the RTEF30 group. They had the highest levels of Enterobacteriaceae (0.4131) and lactic acid bacteria in the early and late phases (0.5556) among the groups. Correlation analysis revealed significant links between *sufu*’s physicochemical properties and microbial communities. Notably, putrescine positively correlated with *Bifidobacterium*, while TA negatively correlated with *Enterococcus*. These findings suggest that microbial activity alterations, caused by low-temperature cold fermentation, influences *sufu*’s fermentation process and quality, guiding further studies on the regulation of biogenic amine formation.

## 1. Introduction

*sufu*, celebrated for its distinctive taste and cultural significance, enjoys a renowned status in the global culinary landscape. Varieties of *sufu*, such as white, green, and red, are distinguished by their post-fermentation hues [1]. The production of *sufu* mainly involves four steps: grinding soybeans, making tofu blocks, cultivating mold, and fermentation. It is one of the four major traditional fermented bean products in China [2]. Soybean fermented products with various physiologically active substances can lower blood pressure, enhance immune function, dissolve blood clots, have anti-aging effects, and reduce cholesterol [3]. Fermented *sufu* is also rich in nutrients essential for normal growth and physiological functions, such as free fatty acids, thiamine, and calcium [4]. It is evident that the various physiologically active substances in *sufu* are closely related to the fermentation process carried out by microorganisms [5]. During the fermentation process, the bitterness and fishy taste of soybeans are removed, while the content of flatulence and anti-nutrient factors are reduced [5]. Additionally, the organic acids, alcohols, and esters produced during fermentation greatly enhance the flavor of *sufu*, making *sufu* both nutritious and delicious.

An increasing number of studies have highlighted the critical role that microorganisms play in the fermentation process of *sufu*. Microorganisms are the backbone of fermented foods, directly influencing factors such as fermentation time, product nutrition, taste, and biosafety [6,7]. During the fermentation process of *sufu*, enzymes such as amylase, protease, and lipase, produced by filamentous fungi and certain bacteria, break down components into smaller, more absorbable nutrients [8]. These small molecules can further metabolize into a variety of volatile compounds, including acids, ketones, esters, alcohols, and amino acids, which significantly contribute to the flavor of *sufu*. Various strains, including *Mucor wutungkiao*, *M. racemosus*, *Rhizopus* spp., and certain bacteria, are introduced during the fermentation of *sufu*. Among these, *Mucor* spp. are the most widely used and distributed, playing a pivotal role in regulating *sufu* quality [5].

The traditional fermentation process of *sufu* involves the addition of edible salt and ethanol, which not only prevent food spoilage but also enhance flavor [9]. As a result, traditional *sufu* is typically characterized by its high-salt content. However, excessive consumption of high-salt foods increases stress on the kidneys, leading to hypertension, and negatively affects overall health [10]. Research on low-salt *sufu* has focused on selecting efficient microbial strains, optimizing fermentation processes, and identifying antimicrobial substances during fermentation. A previous study has demonstrated that low-temperature treatment can suppress spoilage microorganisms in low-salt pickles, ensuring quality and preventing spoilage during storage [11]. Similar studies have also shown that low-temperature fermentation of rice wine maintains key characteristics and significantly improves its aroma without extending fermentation time compared to room-temperature fermentation [12,13]. In fermented foods, low-temperature fermentation offers advantages such as reducing spoilage microorganisms, preserving small-molecule aroma and flavor substances, and preventing the rancidity of lipid components [14]. However, low-temperature fermentation slows down the process, reduces enzyme activities (EAs) related to nutrient biotransformation, and lengthens the fermentation cycle. The maturation of *sufu* relies on microbial proteases to break down proteins into flavorful amino acids and peptides. Therefore, a novel enzyme-assisted low-temperature post-fermentation method (LTEF20) could be used to improve the prolonged fermentation cycles while preserving the advantages of low-temperature fermentation in low-salt-content conditions.

Diet-induced health risks have raised increasing attention on food safety [15]. As important indicators of *sufu* safety, biogenic amines (BAs) can have both beneficial and harmful effects on human health. However, excessive accumulation of BA in the body can result in biogenic amine poisoning, with histamine and tyramine being the most toxic forms [16]. Although putrescine itself has low toxicity, it can enhance the toxicity of histamine and tyramine [17]. Consequently, fermentation techniques aimed at reducing their levels have garnered significant attention from researchers worldwide. They are primarily produced by the decarboxylase from microorganisms such as the *Streptococcus*, *Bacillus*, *Klebsiella*, *Escherichia*, and *Clostridium* genus using free amino acids as substrates [18]. Decarboxylase and other enzymes produced by microorganisms have important impacts on product safety by influencing the formation of BA. Therefore, research on the microecology of *sufu* helps uncover the main functional microbial communities and offers insights into how these communities regulate the quality, flavor, and safety of *sufu*.

Therefore, this study aims to innovatively apply an enzyme-assisted low-temperature fermentation method to produce *sufu* with reduced salt content, thereby mitigating the potential health risks associated with high-salt food consumption [19]. At the same time, this method seeks to shorten the production cycle, addressing the challenges of long fermentation times and the poor stability and controllability found in traditional fermentation processes. Additionally, this study evaluates the physicochemical properties, microbial diversity, and safety regarding BA during the enzyme-assisted low-temperature fermentation process. Through correlation analyses, the role of microorganisms in the fermentation process will be explored, providing valuable insights for the industrial automation, transformation, and upgrading of *sufu* production.

## 2. Materials and Methods

### 2.1. Materials and Reagents

The table salt, brine tofu, and strong-flavored white wine were all food-grade; *Actinomucor elegans* mold powder was purchased from Angel Yeast Co., Ltd. (Yichang, China). The colony texture is cottony. Food-grade alkaline and neutral proteases (activity units of 1 × 10^5^ U), with the optimal temperatures of enzyme activities ranging from 20 °C to 60 °C, were purchased from Xiasheng Enzyme Biotechnology Co., Ltd. (Yinchuan, China). Analytical-grade ammonium sulfate, glacial acetic acid, acetylacetone, 37% formaldehyde, and anhydrous sodium acetate were purchased from Aladdin Industrial Corporation (Shanghai, China).

### 2.2. Enzyme-Assisted Low-Temperature Fermentation of sufu

**Figure 1:**
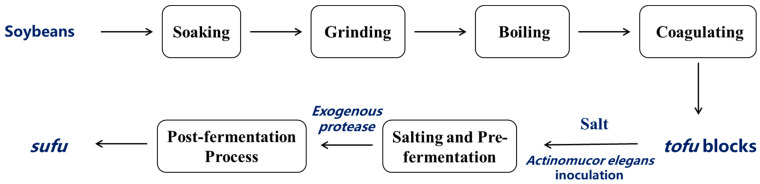
Flowchart of enzyme-assisted *sufu* fermentation process.

As shown in Figure 1, the fermentation process of *sufu* began with the selection of soybeans, which were soaked, ground, and boiled to make tofu. The tofu was then cut into blocks and subjected to the salting process and mold cultivation. During the post-fermentation process, exogenous protease was used to facilitate the breakdown of the proteins and complete the fermentation to produce *sufu*. *Actinomucor elegans* was inoculated onto spore-producing culture medium (bran solid medium composed of 5 g bran and 5 mL distilled water) and incubated at 26 °C for 4 days until the fungal spores had grown sufficiently. Then, 20 mL of sterile water was added to the conical flask containing the seed culture, followed by vigorous shaking for 2–3 min to elute the spores off the surface of the bran solid medium, and they were filtered through three layers of sterile gauze. After counting the obtained fungal spores, the spore suspension was adjusted to a concentration of 1 × 10^7^ CFU/mL using sterile water for a fermentation starter. Commercial brine-aged tofu was cut into even blocks (2 × 2 × 2.5 cm^3^) and evenly sprayed with the prepared spore suspension.

The tofu was pre-fermented at 25 °C in an incubator for 3 days, during which the fungal spores attached to the surface of the fermented tofu, and began to germinate and extend outward, forming thread-like hyphae. The mold cultivation was terminated when the hyphae intertwined to form a network that fully covered the tofu’s surface, creating a ’fuzzy’ layer [20]. Then, salt was added at a 10:1 weight ratio, and the tofu was brined at 25 °C for 22 h to obtain the salted tofu curd. The salted tofu curd was weighed and then divided into 11 portions. Based on previously published studies, the optimal conditions for exogenous protease treatment, a 2% neutral protease + 2% alkaline protease combination, were applied [5]. Finally, the salted tofu curd was completely soaked in 10% edible ethanol, sealed, and fermentation was conducted under three conditions for 20 days: enzyme-assisted fermentation at low temperature (20 °C) (LTEF20), no enzyme fermentation at high temperature (30 °C) (RTNF30), and enzyme-assisted treatment at 30 °C (RTEF30). The samples were collected on days 0-3 of pre-fermentation (Q0, Q1, Q2, and Q3) and at 1, 2, 3, 5, 10, and 20 days post-fermentation under three treatments (S1 for RTNF30, S2 for LTEF20, and S3 for RTEF30), denoted as H1, H2, H3, H5, H10, and H20.

### 2.3. Determination of Physical and Chemical Properties of sufu

The TA content was determined using the sodium hydroxide titration method [21]. A 2.0 mL sample of the *sufu* solution was mixed with 50 mL of deionized water and titrated with a 0.05 mol/L sodium hydroxide solution, using phenolphthalein as the indicator. The results were expressed as the percentage of lactic acid. For reference, the SB/T 10170-2007 *sufu* operation manual was consulted for the measurement of amino nitrogen (NH3-N) content in the sample.

For the detection of EA, a 10.0 g/L casein solution and a 0.4 mol/L trichloroacetic acid solution were added to the sample liquid. The mixture was then centrifuged at 1000 rpm for 3 min. The supernatant was collected, and a 0.4 mol/L sodium carbonate solution followed by Folin’s reagent solution were added. The mixture was heated in a water bath and the absorbance was measured at 680 nm [22].

To determine the BA content, 1 mL of the biogenic amine standard solution or sample was taken into a 5 mL brown colorimetric tube. Sequentially, 2 mol/L NaOH and NaHCO_3_ solutions were added, followed by 10 mg/mL of danisone-pyrrole solution. The sample underwent a cold-water bath for 30 min, after which 100 μL of 25% concentrated ammonia solution was added. The volume was made up with chromatographic grade acetonitrile, and the sample was filtered through a 0.22 μm organic membrane before analysis by high-performance liquid chromatography (HPLC). An Atlantic C18 column (4.6 mm × 250 mm, 5 μm) was used, maintaining the column temperature at 25 °C. The mobile phase consisted of 0.1% formic acid in acetonitrile (A) and 0.1% formic acid in water (B). The elution program was set as follows: 0–7 min, 50–55% B; 7–25 min, 55–90% B; 25–32 min, 90–55% B; 32–38 min, 55–55% B. The injection volume was 20 µL; the flow rate was 1.0 mL/min; the detection wavelength was 254 nm.

### 2.4. Microbiological Diversity Analysis of sufu

High-throughput sequencing technology, based on the bacterial 16S rRNA gene and fungal ITS2 gene, was employed to assess the microbial diversity of *sufu* under various post-fermentation temperatures. This analysis involved clustering and principal component analysis. For each group of enzyme-treated *sufu*, 1.5 g of the sample was used for metagenomic DNA extraction, and the quality of the DNA was evaluated. Upon passing the quality assessment, the samples were submitted to Shanghai Meiji Biotechnology for microbial diversity analysis of bacteria and fungi using the Illumina MiSeq high-throughput sequencing platform.

The bacterial 16S rRNA gene fragments were amplified using the universal primers 338F and 806R, with sequences 5′-ACTCCTACGGGAGGCAGAG-3′ and 5′-GGACTACHVGGGTWTCTAAT-3′, respectively. The sequences were clustered to create operational taxonomic units (OTUs) using UPARSE 7.0 with a similarity of greater than 97%. The microbial diversity and relative abundance of bacteria were analyzed through comparison with database sequences.

### 2.5. Methods of Statistical Analysis

Origin 8.6 software was used for graphing, SPSS 25.0 software was employed for correlation analysis, and the Majorbio cloud platform (available online: https://cloud.majorbio.com/) (accessed on 1 January 2021) was utilized for principal component analysis (PCA), which included statistical analysis and plotting. This ensured that the data were analyzed with precision and displayed professionally. The 16S rRNA sequencing was analyzed by utilizing the Majorbio cloud platform (available online: https://cloud.majorbio.com/) (accessed on 1 January 2021), while other statistical analyses were performed using GraphPad Prism 8 (GraphPad Software Inc.)(San Diego, CA, USA). Group differences were identified using one-way analysis of variance (ANOVA) followed by Tukey’s multiple comparison posttest. All data were shown as mean ± SEM, where a *p*-value of less than 0.05 indicates statistical significance.

## 3. Results and Discussion

### 3.1. Changes in the Physicochemical Properties of sufu

**Figure 2:**
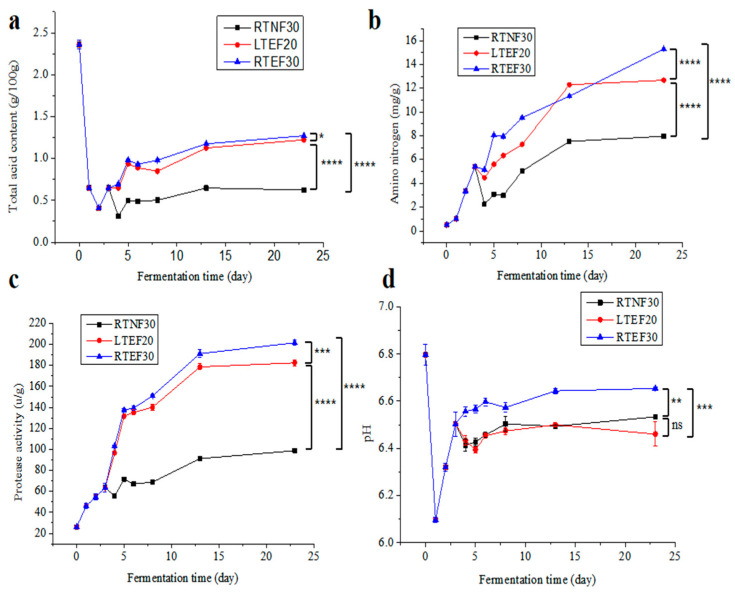
Changes in the physicochemical properties of *sufu* in different post-fermentation conditions: (**a**) total acid contents, (**b**) amino nitrogen contents, (**c**) protease activities, (**d**) pH value. RTNF30, non-enzyme-assisted fermentations at room-temperature. LTEF20, enzyme-assisted fermentations at low-temperature. RTEF30, enzyme-assisted fermentations at room-temperature. * indicates a significant difference at the 0.05 level. ** indicates a significant difference at the 0.01 level. *** indicates a significant difference at the 0.001 level. **** indicates a significant difference at the 0.0001 level. ns indicates no significant difference at the 0.05 level.

The data revealed that the TA content in *sufu* for all the groups initially decreased in the pre-fermentation phase (0–1 day) (Figure 2a), possibly attributed to the consumption of organic acids in Tofu material by the starter microbes. Then, the TA contents increased and then plateaued in pre-fermentation phase (2–3 day) and post-fermentation phase (4–23 day), mainly due to the generation of small molecules such as peptides and amino acids by microbes. In the late post-fermentation phase, microbes consumed some organic acids, leading to a decrease in TA contents [23]. The TA content in the LTEF20 and RTEF30 groups was significantly higher than that in the RTNF30 group, indicating high activities of microbes in the enzyme-assisted fermentation groups. In Figure 2d, the pH value dropped sharply in the early stage of fermentation and gradually increased to a certain level. However, this result seemed to differ from the TA changes (Figure 2a). The reason for this difference was possibly related to the varying effects of different organic acids on pH [24].

Figure 2b,c show that the NH3-N content and EA in all experimental groups exhibited a trend of first increasing and then leveling off. Among them, the NH3-N content and EA in the LTEF20 and RTEF30 groups were significantly higher than those of the RTNF30 group, indicating that the enzyme-assisted fermentation accelerated the maturation of *sufu*. Under enzyme-added conditions (LTEF20 and RTEF30), higher temperatures correlated with increased NH3-N content (Figure 2b), but the low-temperature post-fermentation strategy had advantages in controlling acid spoilage and enhancing food biosafety [25]. Nevertheless, lower temperatures would result in a decrease in microbial enzyme production and enzyme activity, which extended the fermentation period. It was found that LTEF20 had a maturation rate similar to the RTEF30 group in Figure 2b. This suggested that the addition of exogenous protease could effectively address the problems of a prolonged fermentation period under low-temperature conditions. Consistent with the NH3-N content, all the groups showed escalated EAs as fermentation progressed (Figure 2c). In the pre-fermentation stage (0–3 days), EAs increased as microorganisms rapidly proliferated and produced enzymes to degrade nutrients for their growth. During the post-fermentation phase (4–23 days), the metabolisms of fungi were inhibited and the increased enzymes were mainly produced by those bacteria tolerated in a high-salt environment. But the high-salt stress led to a slower increase in protease production until the end of fermentation [9,26]. In addition, the protease activities in the LTEF20 and RTEF30 groups were markedly higher compared to those in the RTNF30 group, indicating the viability of the enzyme-assisted low-temperature fermentation strategy.

### 3.2. Changes in the Biogenic Amine Content of sufu

**Figure 3:**
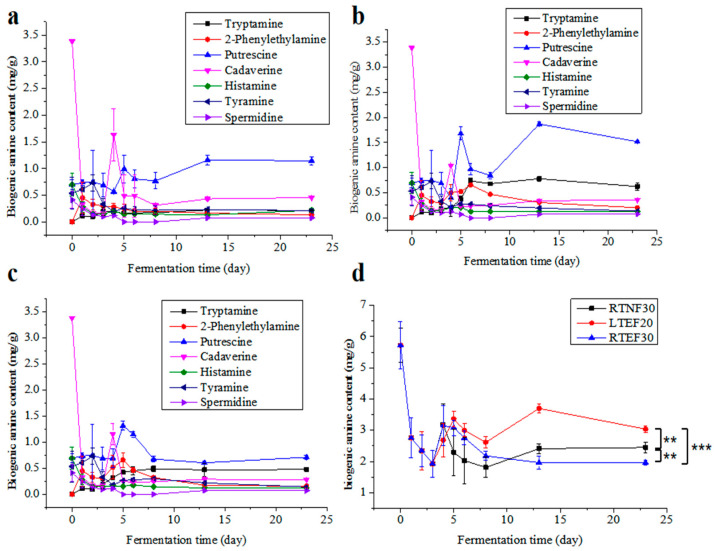
Comparison of biogenic amine contents in different groups. (**a**) Non-enzyme-assisted fermentations at room-temperature (RTNF30) control group; (**b**) enzyme-assisted fermentations at low-temperature group (LTEF20); (**c**) enzyme-assisted fermentations at room-temperature group (RTEF30); (**d**) comparison of total biogenic amines in different groups. ** indicates a significant difference at the 0.01 level. *** indicates a significant difference at the 0.001 level.

Biogenic amines (BAs) were recognized for their multiple roles, where they could stimulate the synthesis of DNA, RNA, and proteins; expedite growth and development, regulate blood pressure; and neutralize free radicals. However, excessive consumption of BA was associated with health problems such as diarrhea, abdominal pain, headaches, and even life-threatening poisoning [16,27]. Therefore, it was imperative to meticulously manage BA levels during the fermentation of *sufu*. Analysis of Figure 3a–c revealed that the levels of cadaverine, histamine, tyramine, and spermidine in all the groups remained relatively stable during the whole fermentation period. In contrast, the levels of tryptamine, phenylethylamine, putrescine, and total BA initially increased, and then decreased, generally peaking on the 14th day of fermentation. Among these, putrescine was the most abundant, while spermidine was the least.

The data suggested that the TBA content exhibited a pattern of initial increase followed by a plateau throughout the post-fermentation process (Figure 3d). This trend could be explained by the low microbial content and limited amino acid decarboxylase activity observed at the onset of fermentation, and a rapid proliferation of microorganisms subsequently, leading to an elevation in amino acid decarboxylase levels and a surge in BA content. Eventually, the production and consumption of BA by microbes reached a dynamic balance. Histamine was acknowledged as the most toxic of the known BAs. It was reported that elevated levels of phenylethylamine and tryptamine were linked to increased blood pressure [28]. In all the groups, the levels of histamine and putrescine were comparable and below the established BA risk thresholds. On day 20 of the post-fermentation period, putrescine, cadaverine, and tryptamine constituted a large portion of the TBA present. Putrescine and cadaverine exacerbated the toxicity of histamine by suppressing the activity of histamine-metabolizing enzymes and methyltransferases [16,29], and they also served as precursors to the potent carcinogen nitrosamines.

The enzyme-added groups (RTEF30 and LTEF20) exhibited higher total BA levels than the RTNF30 group, and the LTEF20 showed significantly higher total BA levels than RTEF30 (Figure 3d). This suggested that temperature significantly affected the production of BA [30]. The findings of this study indicated that although the BA content increased marginally under low-temperature conditions, the levels remained well below the existing safety thresholds for fermented soybean products.

### 3.3. Correlation Analysis Between Different Physicochemical Properties and BA

It was indicated that the introduction of exogenous proteases and the cold post-fermentation technology could influence the fluctuations in TBA content. Therefore, this BA alteration is potentially linked to physicochemical properties such as NH3-N and microbial fermentation activities. The Pearson correlation analysis presented in Table 1 revealed that total BA possessed a significant correlation with cadaverine, histamine, tyramine, and spermidine, with the strongest correlation being with histamine (r = 0.773, *p* < 0.01). In addition, putrescine, cadaverine, histamine, tyramine, spermidine, and tryptamine exhibited a strong correlation each other. Among these, cadaverine and histamine displayed the highest correlation coefficient (r = 0.864, *p* < 0.01), followed by spermidine and histamine (r = 0.856, *p* < 0.01). Additionally, histamine and tyramine also demonstrated a significant correlation. Research had indicated that guanidino-butylamine could synthesize putrescine, and both histamine and tyramine could influence the synthesis of guanidino-butylamine, thereby affecting the synthesis of putrescine [31].

The analysis revealed that TA had a significant positive correlation with spermidine and cadaverine, and an even closer relationship with histamine. *sufu* was fermented in a weakly acidic environment, where microorganisms underwent decarboxylation reactions to produce alkaline BA, as a means to counteract the acidic environment [32]. Consequently, the low TA content under natural fermentation conditions also indicated a lower biogenic amine content in *sufu* under these circumstances. The NH_3_-N content exhibited a significant positive correlation with cadaverine, histamine, and spermidine, and an extremely significant positive correlation with tryptamine and tyramine, suggesting that the maturation process of *sufu* was tightly associated with the production of BA. The protease activity content exhibited a significant positive correlation with cadaverine and spermidine, and an extremely significant positive correlation with tryptamine, his, and tyramine. This further substantiated the close relationship between amino acid nitrogen content and the formation of BA. Therefore, it is very necessary to fully understand the potential health risks and establish and reach safety standards associated with these foods to reduce health hazards [25].

### 3.4. The Impact of LTEF20 on Bacterial Diversity at Different Fermentation Stages

In this study, high-throughput sequencing was employed to track changes in microbial diversity throughout the *sufu* fermentation process. Bacterial diversity was assessed under various conditions and at different stages. The research concentrated on alpha diversity to assess microbial richness and diversity, with the Chao and ACE indices serving as key richness metrics. As shown in Table 2, all samples had a sequencing coverage index above 0.999, signifying high detection of *sufu* sequences and reliable results [33]. The Chao and ACE indices revealed that bacterial community abundance was highest during the low-temperature enzyme-added post-fermentation (LTEF20) stage and increased with fermentation time. S2H10 exhibited exceptionally high ACE and Chao values, suggesting high species richness. The Shannon and Simpson indices indicated a more even distribution in the LTEF20 group, whereas the room-temperature non-enzyme-added group had the lowest Shannon and highest Simpson values, suggesting dominance by a few species.

**Figure 4:**
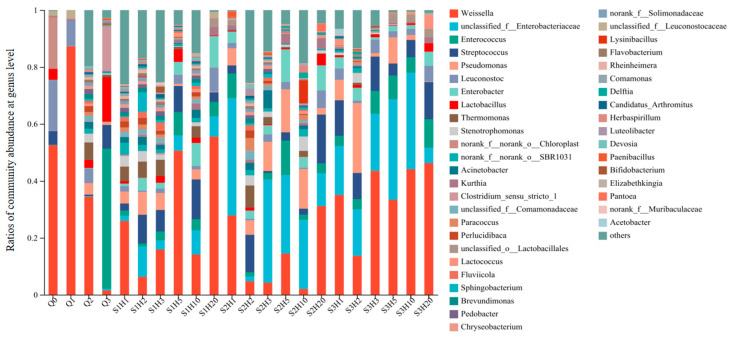
Bacterial community abundances of sufu samples during fermentation. Q0, Q1, Q2, and Q3 indicate the pre-fermentation at 0, 1, 2, and 3 days. S1H1, S1H2, S1H3, S1H5, S1H10, and S1H20 indicate the post-fermentation of RTNF30 at 1, 2, 3, 5, 10, and 20 days. S2- indicates the post-fermentation of LTEF20, and S3- indicates post-fermentation of RTEF30 at different days.

Microbial composition and abundance in *sufu* samples varied across different stages and treatments. Figure 4 illustrates the bacterial community structure at the genus level for each *sufu* sample, highlighting species with a relative abundance greater than 0.1%. The *x*-axis indicates the *sufu* samples under various fermentation conditions and periods, while the *y*-axis shows the relative abundance of different bacterial genera in each sample, clearly delineating the similarities and differences in microbial community composition among samples. A total of 831 bacterial genera were identified across 22 samples, with only 43 genera having a relative abundance above 0.1%. It was clearly demonstrated that the community structure fluctuated over time under different fermentation conditions, with shifts in bacterial species and their relative proportions. The six bacterial genera with the highest average abundance throughout the fermentation process were *Weissella*, *Streptococcus*, *Pseudomonas*, *Leuconostoc*, *Enterococcus*, and *Enterobacter*. The *Enterobacteriaceae* family predominated during post-fermentation days 3, 5, and 10, while *Weissella* was most abundant in both the early and late stages of fermentation.

The differences in bacterial species under various fermentation times and conditions reflected the ability to control microbial species and quantities by regulating fermentation time and conditions. At the genus level, *Weissella* had the highest relative abundance in the pre-fermentation stage, in both the room-temperature enzyme-added and room-temperature non-enzyme-added groups. During the post-fermentation stage, lactic acid bacteria were the predominant constituents of the bacterial communities in both RTNF30 and RTEF30 goups. Conversely, in the LTEF20 group, the genus *Enterobacter* was predominantly dominant, followed by other lactic acid bacteria. These findings indicated that low-temperature fermentation processes differed from traditional room-temperature methods in the different microbial compositions and abundances, which could lead to significant changes in the quality and flavor of *sufu*.

Statistical analysis of *Weissella* indicated that the abundance percentage of *Weissella* was 0.84% in the LTEF20 group, which was significantly lower compared to the RTNF30 group (1.68%) and RTEF30 group (2.16%). Additionally, although microbial diversity varied significantly among the three treatments during fermentation, it tended to stabilize by the end of the fermentation process (post-fermentation day 20), with a high presence of *Weissella* across all groups. This suggested that lactic acid bacteria (LBA), particularly *Weissella*, played a crucial role in the maturation process of *sufu*. Liang, T. et al. found that the major genera during *sufu* fermentation included *Lactobacillus* and *Weissella*, which is consistent with the finding in this study [34]. The genus *Weissella* was identified in various fermented foods, including pickles, liquor, and soy sauce. *Weissella* not only conferred a distinctive flavor to these products but also provided probiotic advantages, inhibited spoilage microorganisms, and suppressed certain pathogenic bacteria [35]. Investigations into various soy-based fermented foods disclosed that the predominant microbial genera in these samples were *Bacillus* and *Weissella* [36]. In addition, other LAB, *Lactococcus* and *Streptococcus*, were found to be abundant in different *sufu* fermentation process. The growth of *Lactococcus* not only extended the shelf life of fermented bean curd but also successfully suppressed harmful pathogens. The substantial presence of *Lactococcus* in the bean curd and the production of gamma-aminobutyric acid by *Lactococcus lactis* significantly enhanced the physiological properties of *sufu* [37]. Furthermore, it was established that *Lactococcus* effectively curtailed the growth of pathogenic bacteria, such as *Staphylococcus aureus* [38].

**Figure 5:**
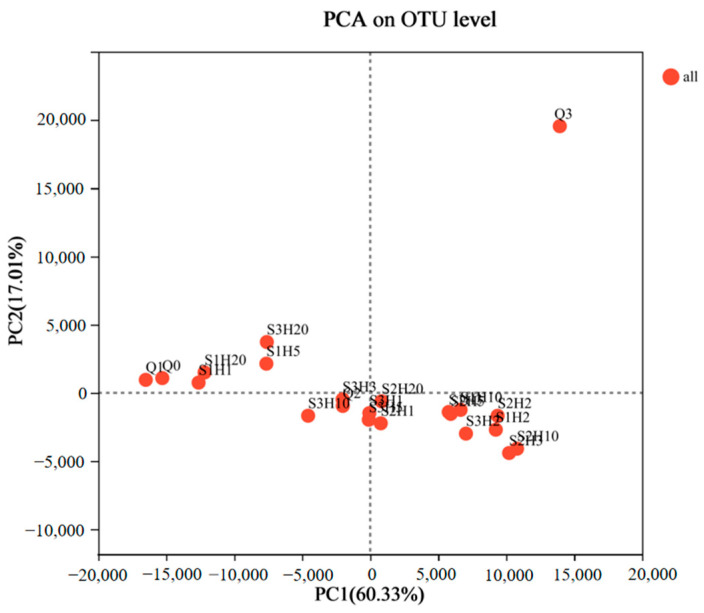
Principal component analysis (PCA) of microbial OTU abundances of sufu samples during fermentation. OUT, operational taxonomic units. Q0, Q1, Q2, and Q3 indicate pre-fermentation at 0, 1, 2, and 3 days. S1H1, S1H2, S1H3, S1H5, S1H10, and S1H20 indicate post-fermentation of RTNF30 at 1, 2, 3, 5, 10, and 20 days. S2- indicates post-fermentation of LTEF20, and S3- indicates post-fermentation of RTEF30 at different days.

The Principal Component Analysis (PCA) results for various samples are depicted in Figure 5. The first and second principal components (PC1 and PC2) explained 60.33% and 17.01% of the bacterial community variance, respectively. This indicated that the combined PC1 and PC2 values, totaling 77.34%, effectively captured the species variation among the samples. Pre-fermentation samples Q0, Q1, and Q3 displayed minimal similarity in bacterial diversity and composition compared to the post-fermentation samples. This suggested significant differences in bacterial communities between the pre- and post-fermentation stages, likely due to distinct fermentation conditions, oxygen levels, and chemical components such as ethanol. The later stage of pre-fermentation (Q3) showed increased fungal growth, including *Mucor* species, along with the influence of enzymes and metabolites, leading to substantial differences from the earlier pre-fermentation stages (Q0 and Q1).

The PCA plot further revealed that the remaining 18 samples could be categorized into three distinct clusters: S1H1, S1H20, S1H5, and S3H20 formed one group with high similarity. S3H10, S3H3, S2H1, S3H5, S3H1, and S2H20 constituted a second group. S1H3, S2H5, S1H10, S1H2, S2H2, S2H3, and S2H10 made up the third group. This indicated that while similarities and differences existed in bacterial communities across various fermentation stages and conditions, the fermentation temperature significantly impacted the microbial diversity in the final product. Compared to the 20 °C post-fermentation samples (S2), the microbial diversity of the 30 °C post-fermentation samples (S1 and S3) were more similar at the fermentation endpoint (20 days), indicating that temperature was a critical factor influencing microbial growth and the quality of the fermented product. However, the pre- and early-stage post-fermentation (within 10 days) phase showed some similarities between the S1 and S2 treatments, suggesting that factors such as raw materials also influenced microbial growth. Moreover, the PCA indicated that enzyme-treated post-fermentation samples (S2 and S3) exhibited greater microbial diversity similarity compared to the non-enzyme treated post-fermentation samples (S1). This suggested that the addition of proteases could alter the protein degradation rate in the fermentation substrate, indirectly affecting microbial growth and the characteristics of the final product. Liu, X. et al. reported that during fermentation for L-glutamate production, fermentation efficiency and microbial composition were significantly improved by adding exogenous protease to the fermentation broth [39]. This confirmed the important effect of the exogenous protease on the microbial composition in fermentation. The microbial diversity of the post-fermentation samples after 10 days showed minimal changes, but after 20 days, the microbial diversity across all treatments showed a trend toward similarity, with a high *Weissella* content in all samples. The differences in microbial diversity among various post-fermentation treatments highlighted the complex mechanisms in fermented tofu fermentation [40]. Therefore, further correlation analysis was conducted to explore the impact of specific microbial communities on biogenic amines and other physicochemical properties.

### 3.5. Roles of Microbial Diversity in Influencing Physicochemical Properties and Biogenic Amines

**Figure 6:**
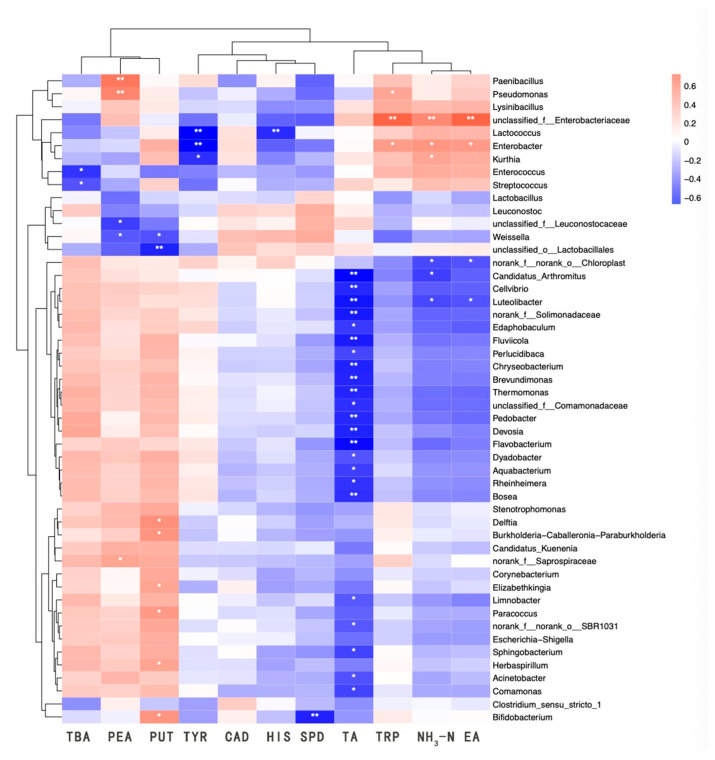
Correlation heatmap analysis between microbial communities and physicochemical properties and biogenic amines. BA, bioamine acid. TA, total acid. NH3-N, amino nitrogen. EA, enzyme activity. * indicates a significant correlation at the 0.05 level; ** indicates a significant correlation at the 0.01 level.

Biogenic amines (BAs) generated during fermentation primarily originated from the decarboxylation of amino acids by microorganisms. Consequently, a correlation existed between the microbes and the content of BA during fermentation. Microorganisms such as LAB, *Pseudomonas,* and *Enterobacter*, exhibiting decarboxylase activity, are the main amine-producing bacteria in traditional fermented foods [41]. This is consistent with the findings of the present study. The heatmap depicting the correlation between microorganisms and physicochemical parameters is displayed in Figure 6. Phenylethylamine was significantly and positively correlated with *Paenibacillus* and *Pseudomonas*, and moderately correlated with *Saprospiraceae*. Tryptamine was significantly and positively correlated with Enterobacteriaceae, and moderately correlated with *Enterobacter* and *Pseudomonas.* Putrescine exhibited a significant positive correlation with *Bifidobacterium*, *Elizabethkingia*, *Burkholderia-Caballeronia-Paraburkholderia*, *Herbaspirillum*, *Delftia*, and *Paracoccus*. Additionally, 2-phenylethylamine displayed a significant negative correlation with *Weissella* and *Leuconostocaceae*, while putrescine showed a significant negative correlation with *Weissella* and a highly significant negative correlation with *Lactobacillaceae*. Tyramine demonstrated a highly significant negative correlation with *Lactococcus* and *Enterobacter*, and a significant negative correlation with *Kurthia*. Histamine exhibited a highly significant negative correlation with *Lactococcus*, and spermidine showed a highly significant negative correlation with *Bifidobacterium*. Lastly, TBA revealed a significant negative correlation with Streptococcus and *Enterococcus*.

Pseudomonas was renowned for its protein-degrading capabilities and was commonly found in protein-rich foods such as meats and beans [42]. Its salt tolerance explained its ability to survive in *sufu*, but excessive proliferation could lead to health issues like diarrhea. *Pseudomonas* could also contribute to the accumulation of biogenic amines (BAs) by increasing the fatty acid content within the system, while the *Bacillus* genus might suppress amine-producing bacteria through the production of bacteriocins. As a result, amino acid nitrogen could promote the growth of *Pseudomonas* [43]. The physicochemical properties of the samples influenced microbial physiological activity, which affected the levels of BA in food. Therefore, both the physicochemical properties and the microbial community played significant roles in the formation of BA.

Figure 6 demonstrates that numerous genera exhibited negative correlations with TA content. Enterobacteriaceae and *Enterobacter* were positively correlated with NH3-N and protease activity, likely due to their protease production or promotion by amino compounds, while *Luteolibacter* showed a negative correlation with both NH3-N and EA. A significant negative correlation was observed between TA content and various microorganisms, allowing the microbial community to be broadly categorized into two groups: one that was negatively correlated with TA and another that was insensitive to TA. This implied a strong association between the acidic environment and microbial composition, as well as metabolic types. The group of microorganisms negatively correlated with TA displayed a positive correlation with the primary BA constituents of *sufu*, such as phenylethylamine, putrescine, and TBA content. *Acinetobacter* species were significantly positively correlated with putrescine, negatively correlated with cadaverine and histamine, though not significantly, and significantly negatively correlated with TA content. They also showed a negative correlation with NH3-N and EA, but not significantly. The *Enterobacter* group within the TA-insensitive clade significantly influenced the levels of cadaverine, EA, and NH3-N components, which were crucial for *sufu* production and likely played a pivotal role in it. Some members of this clade notably inhibited the production of major BA constituents, suggesting that these microorganisms could have contributed to the safety of *sufu* by curbing the growth of BA through microbial interactions and metabolites. The intricate relationships between microorganisms and physicochemical properties indicated that further research was necessary to elucidate the connections between strains, BA, and physicochemical properties.

## 4. Conclusions

This study evaluated the feasibility and safety of low-temperature enzyme-assisted fermentation of *sufu*. The study indicates that LTEF20 is viable based on physicochemical properties, and biogenic amine levels, suggesting the acceptability of low-temperature enzyme-assisted *sufu* in terms of biogenic amine risk. Bacterial diversity analysis revealed differences in microbial communities under various fermentation conditions, with an increase in species richness and evenness in the LTEF20 group. Correlation analysis between physicochemical properties and BA contents showed connections between TA, protease activity, and BA, with lower TA content under low-temperature conditions, corresponding to lower biogenic amine levels. High-throughput sequencing technology revealed differences in microbial communities under different fermentation conditions, with Enterobacteriaceae and *Weissella* being dominant under low-temperature enzyme-assisted fermentation, differing from traditional fermentation. These differences impacted the quality and safety of *sufu*. In conclusion, this study provides scientific evidence for the potential benefits of the enzyme-assisted low-temperature post-fermentation process, contributing to the improvement of *sufu* quality and safety. However, several issues remain to be addressed in practical applications. Future research should focus on optimizing the fermentation process, studying microbial communities, controlling biogenic amine content, evaluating quality and safety, and promoting industrial applications, to further promote the sustainable development of the *sufu* industry.

## Figures and Tables

**Table 1:** Correlation analysis between all biogenic amines and different physicochemical properties.

Index	Tryptamine	2-Phenylethylamine	Putrescine	Cadaverine	Histamine	Tyramine	Spermidine	Total BA	TA	NH3-N	EA
Tryptamine	1	0.377	0.485 *	0.438 *	−0.486 *	−0.482 *	−0.506 *	−0.349	0.198	0.737 **	0.857 **
2-Phenylethylamine		1	0.142	−0.354	−0.319	0.016	−0.343	−0.225	−0.272	−0.078	0.162
Putrescine			1	−0.309	−0.178	−0.215	−0.248	−0.040	0.151	0.395	0.409
Cadaverine				1	0.864 **	0.179	0.767 **	0.746 **	0.530 *	0.470 *	0.467 *
Histamine					1	0.486 *	0.856 **	0.773 **	0.655 **	0.505 *	0.500 **
Tyramine						1	0.559 **	0.442 *	0.076	0.561 **	0.563 **
Spermidine							1	0.745 **	0.521 *	0.427 *	0.473 *
Total BA								1	0.367	0.597 **	0.582 **
TA									1	0.250	0.274
NH3-N										1	0.932 **
EA											1

Note: BA, bioamine acid. TA, total acid. NH3-N, amino nitrogen. EA, enzyme activity. * indicates a significant correlation at the 0.05 level. ** indicates a significant correlation at the 0.01 level.

**Table 2:** α-Diversity Index comparison of sufu samples during fermentation.

Samples\Estimators	Ace	Chao	Shannon	Simpson
Q0	203.758	153.813	1.533	0.330
Q1	41.881	40.250	0.553	0.765
Q2	394.580	398.240	3.597	0.130
Q3	286.834	286.324	2.001	0.285
S1H1	574.101	588.071	4.209	0.077
S1H2	388.527	390.500	4.112	0.036
S1H3	435.946	439.125	4.457	0.040
S1H5	346.094	343.875	2.440	0.274
S1H10	416.670	421.848	3.944	0.054
S1H20	125.908	100.000	1.890	0.316
S2H1	173.680	137.353	2.323	0.163
S2H2	476.776	479.531	4.414	0.034
S2H3	519.401	520.520	3.698	0.079
S2H5	642.061	638.676	2.948	0.091
S2H10	910.278	910.357	4.162	0.051
S2H20	255.716	257.000	2.701	0.145
S3H1	309.180	325.125	2.740	0.161
S3H2	313.308	315.000	3.479	0.071
S3H3	289.938	305.000	2.331	0.231
S3H5	74.647	73.667	2.320	0.171
S3H10	76.739	83.000	2.031	0.247
S3H20	106.012	103.500	2.222	0.229

Notes: Q0, Q1, Q2, and Q3 indicate the pre-fermentation at 0, 1, 2, and 3 days. S1H1, S1H2, S1H3, S1H5, S1H10, and S1H20 indicate the post-fermentation of RTNF30 at 1, 2, 3, 5, 10, and 20 days. S2- indicates the post-fermentation of LTEF20, and S3- indicates post-fermentation of RTEF30 at different days.

## Data Availability

The original contributions presented in this study are included in the article. Further inquiries can be directed to the corresponding author.
